# Trends in survival of chronic lymphocytic leukemia patients in Germany and the USA in the first decade of the twenty-first century

**DOI:** 10.1186/s13045-016-0257-2

**Published:** 2016-03-22

**Authors:** Dianne Pulte, Felipe A. Castro, Lina Jansen, Sabine Luttmann, Bernd Holleczek, Alice Nennecke, Meike Ressing, Alexander Katalinic, Hermann Brenner, GEKID Cancer Survival Working Group

**Affiliations:** Division of Clinical Epidemiology and Aging Research, German Cancer Research Center (DKFZ), Im Neuenheimer Feld 581, 69121 Heidelberg, Germany; Cardeza Foundation and Division of Hematology, Department of Medicine, Thomas Jefferson University, Philadelphia, PA USA; Bremen Cancer Registry, Leibniz-Institute for Prevention Research and Epidemiology—BIPS, Bremen, Germany; Saarland Cancer Registry, Saarbrücken, Germany; Hamburg Cancer Registry, Authority for Health and Consumer Protection, Hamburg, Germany; Cancer Registry of Rhineland-Palatinate, Institute for Medical Biostatistics, Epidemiology and Informatics, University Medical Center, Johannes Gutenberg University Mainz, Mainz, Germany; Cancer Registry of Schleswig-Holstein, Lübeck, Germany; Division of Preventative Oncology, German Cancer Research Center (DKFZ), Heidelberg, Germany; German Cancer Consortium (DKTK), German Cancer Research Center (DKFZ), Heidelberg, Germany

## Abstract

**Background:**

Recent population-based studies in the United States of America (USA) and other countries have shown improvements in survival for patients with chronic lymphocytic leukemia (CLL) diagnosed in the early twenty-first century. Here, we examine the survival for patients diagnosed with CLL in Germany in 1997–2011.

**Methods:**

Data were extracted from 12 cancer registries in Germany and compared to the data from the USA. Period analysis was used to estimate 5- and 10-year relative survival (RS).

**Results:**

Five- and 10-year RS estimates in 2009–2011 of 80.2 and 59.5 %, respectively, in Germany and 82.4 and 64.7 %, respectively, in the USA were observed. Overall, 5-year RS increased significantly in Germany and the difference compared to the survival in the USA which slightly decreased between 2003–2005 and 2009–2011. However, age-specific analyses showed persistently higher survival for all ages except for 15–44 in the USA. In general, survival decreased with age, but the age-related disparity was small for patients younger than 75. In both countries, 5-year RS was >80 % for patients less than 75 years of age but <70 % for those age 75+.

**Conclusions:**

Overall, 5-year survival for patients with CLL is good, but 10-year survival is significantly lower, and survival was much lower for those age 75+. Major differences in survival between countries were not observed. Further research into ways to increase survival for older CLL patients are needed to reduce the persistent large age-related survival disparity.

## Background

Chronic lymphocytic leukemia (CLL) is a relatively common form of leukemia with a varied prognosis, with some patients having the condition without significant pathology and requiring no treatment for many years while others rapidly require treatment [1]. A number of clinical markers and patient characteristics are used to determine when a patient would need treatment and what type of treatment is offered [2].

Survival for patients with CLL has been documented to have improved on a population level between the 1980s and early twenty-first century in the United States of America (USA) [3]. Several recent publications have examined survival for patients with CLL in Europe [4–8], including an analysis of survival of patients with hematologic malignancies in Germany [7], but data on recent changes in survival for patients with CLL by detailed age groups and gender are lacking.

In 2008, a collaborative project between the German Cancer Research Center (DKFZ) and population-based cancer registries throughout Germany was formed in order to provide comprehensive monitoring of cancer survival throughout Germany [9, 10]. In this study, we provide up-to-date and detailed (age and sex specific) relative survival estimates of German CLL patients based on data from 12 population-based cancer registries, covering 28.05 million inhabitants. We compare the results to data obtained from the Surveillance, Epidemiology, and End Results (SEER) database in order to provide an international comparison using a widely accepted database of known high quality [11].

## Methods

### Data sources

A detailed description of the cancer registries from which data were obtained has been previously published [9, 10]. Briefly, data extracted from 12 cancer registries throughout Germany were included. The 12 cancer registries together provide data from a base population of 28.05 million people. Patients aged 15 or older diagnosed with a first malignant tumor in 1997–2011 with a follow-up date through December 2011 were initially identified. Patients with the ICD-10 code C91.1 (CLL) were selected for analysis.

In order to compare population level survival for CLL in Germany with survival in the USA, data from the SEER13 database were analyzed [11]. The same inclusion criteria as for patients from the German cancer registries were applied for the same time period. The SEER13 database includes data from 13 regional cancer centers in the USA, covering a total population of about 41.5 million people. Centers are chosen for inclusion based on their high quality and epidemiologically interesting population groups. The SEER population is considered to be similar to the general US population with respect to most sociodemographic characteristics, although it may be more affluent than average and may have slightly higher-than-average survival for some cancers [12].

### Statistical methods

Five- and 10-year relative survival estimates for the time period 2009–2011 were calculated using period analysis. Period analysis provides the most up-to-date long-term survival estimates and has been shown to closely predict long-term survival of patients diagnosed in the period of interest (here, 2009–2011) [13, 14]. Age-adjusted survival estimates were derived by computing weighted sums of age-specific survival estimates, using weights according to the International Cancer Survival Standard [15] (15–44, 45–54, 55–64, 65–74, and 75+). Because survival for patients with CLL may vary by age and gender, survival was examined by major age groups as above and by gender as well.

In order to minimize the effect of possible differences in the risk of non-CLL-related death on the comparisons, relative survival was calculated. Relative survival was calculated as the ratio of actual survival to expected survival. Expected survival was estimated according to the Ederer II method [16] using life tables stratified by age, sex, and calendar year obtained from the Federal Statistical Office for Germany and from life tables stratified by age, sex, race, and calendar year for the USA available from the Center for Disease Control and Prevention (CDC) [17]. Differences in 5- and 10-year relative survival between patients in Germany and the USA were tested for statistical significance, overall and by single age groups, using model-based period analysis [18]. In model-based analyses, numbers of deaths were modelled as a function of period of follow-up, age group, and country by Poisson regression with the logarithm of the person-months at risk as offset.

To assess recent trends in survival, 5-year age-standardized relative survival was additionally estimated for the time periods 2003–2005, 2006–2008, and 2009–2011 using modelled period analysis, and trends over time were tested for statistical significance by modelled period analysis. All calculations were carried out using SAS software (version 9.2, SAS, Carey, NC, USA), using macros developed for period and modelled period analysis [18, 19]. Statistical significance was defined by *p* values of <0.05.

## Results

Overall, 22,257 cases of CLL were identified in the German registries and 24,771 cases in the SEER database in 1997–2011 (Table 1). Median age at diagnosis was 69 in Germany and 71 in the USA for all patients. For women, the median age at diagnosis was 71 and 73 in Germany and the USA, respectively. For men, the median ages were 68 and 70, respectively, for Germany and the USA (Table 1). The proportion of cases notified to the registry by death certificate only (DCO) for Germany was 15.6 % and for the USA was 1.4 %. These patients were excluded from the analysis because their survival time was unknown.

**Table 1:** Number of cases and characteristics in Germany and the USA for the period 1997–2011

Country	Source population in 2011 (million)			Total cases	Median age at diagnosis	Microscopically confirmed (%)
Germany						
Total	28.05			22,257	69	87.7
Gender						
Male				12,854	68	88.1
Female				9403	71	87.2
Age groups						
15–44				452	NA	89.1
45–54				1639	NA	89.9
55–64				4350	NA	90.2
65–74				7518	NA	88.0
75+				8298	NA	84.9
USA						
Total	41.5			24,771	71	89.4
Gender						
Male				14,831	70	89.6
Female				9940	73	89.1
Age groups						
15–44				511	NA	94.5
45–54				2275	NA	91.9
55–64				5105	NA	90.2
65–74				6636	NA	90.4
75+				10,244	NA	87.5

*NA* not applicable

Overall, 5-year-age-adjusted survival for patients with CLL in Germany was 77.8 % in 2003–2005 and 80.2 % in 2009–2011 (*p* = 0.004) (Table 2). When analysis by gender was performed, there was a small but statistically significant increase in survival for both men and women at +2.9 and +2.0 % units, respectively, (*p* = 0.04) with persistent survival advantages for women throughout the periods of investigation. Age-specific survival showed higher 5-year relative survival estimates in 2009–2011 for younger patients, ranging from 93.4 % for ages 15–44 to 65.1 % for age 75+. A pattern of slightly increasing survival was observed for all age groups except for 55–64, although the increase was statistically significant for patients age 75+ only, with survival estimates of 62.3 % in 2003–2005 and 65.1 % in 2009–2011 (*p* = 0.03).

**Table 2:** Trend analysis of 5-year-period relative survival for age-group-specific and age-adjusted CLL in Germany and the USA

Variable	2003–2005		2006–2008		2009–2011		*p* Value	Difference
	Relative survival	SE	Relative survival	SE	Relative survival	SE		
Germany								
All^a^	77.8	1.0	80.5	0.8	80.2	0.8	0.004	+2.4
Male^a^	75.5	1.5	80.5	1.2	78.4	1.0	0.04	+2.9
Female^a^	80.9	1.4	80.9	1.1	82.9	1.1	0.04	+2.0
Age group								
15–44	90.9	3.4	94.0	2.5	93.4	2.5	0.7	+2.5
45–54	87.2	2.2	93.0	1.6	91.5	1.6	0.1	+4.3
55–64	86.5	1.4	86.4	1.3	85.9	1.3	0.9	−0.6
65–74	79.5	1.6	80.4	1.3	81.7	1.2	0.06	+2.2
75+	62.3	2.7	67.6	2.1	65.1	1.9	0.03	+2.9
USA								
All^a^	80.7	0.8	83.8	0.7	82.4	0.7	0.1	+1.7
Male^a^	79.7	1.1	82.0	1.0	81.4	1.0	0.2	+1.7
Female^a^	82.6	1.2	86.4	1.0	84.2	1.0	0.4	+1.6
Age group								
15–44	89.9	2.8	90.4	3.1	91.4	3.1	0.8	+1.5
45–54	87.3	1.7	94.0	1.3	95.1	1.2	0.0004	+7.8
55–64	87.6	1.3	89.1	1.2	88.7	1.1	0.5	+1.1
65–74	82.6	1.5	86.0	1.3	83.2	1.3	0.9	+0.6
75+	68.4	1.8	71.6	1.7	69.3	1.6	0.7	+0.9

*p* value for trend, derived from the model. Diff = change from 2003–2005 to 2009–2011

*SE* standard error

^a^Age adjusted

For patients in the USA, overall age-adjusted 5-year survival estimates were 80.7 % in 2003–2005 and 82.4 % in 2009–2011 (*p* = 0.1). Analysis by gender likewise showed higher survival among women than among men, with slight, statistically not significant increases in survival over time for both men and women (Table 2). When age-specific analysis was performed, a strong, statistically significant increase in survival was observed for ages 45–54, with an increase of 7.8 % units between 2003–2005 and 2009–2011 (*p* = 0.0004). In the other age groups, much more subtle, statistically not significant increases were observed.

Direct comparison between survival in Germany and the USA was made (Table 3). Five-year relative survival estimates were similar for Germany and the USA in 2009–2011, both overall and by gender- and age-specific analyses. However, a trend towards better survival was observed for patients in the USA at all ages except for age 15–44.

**Table 3:** Five- and 10-year-period relative survival for patients with CLL in 2009–2011

	5-year RS (SE) Germany			5-year RS (SE) USA	Difference		*p* (country)	10-year RS (SE) Germany			10-year RS (SE) USA	Difference			*p* (country)
All^a^	80.2 (0.8)			82.4 (0.7)	−2.2		0.09	59.5 (1.3)			64.7 (1.1)	−5.2			0.0001
Gender															
Male^a^	78.4 (1.0)			81.4 (1.0)	−3.0		0.07	57.4 (1.8)			63.0 (1.5)	−5.6			0.0001
Female^a^	82.9 (1.1)			84.2 (1.0)	−1.3		0.7	63.6 (1.8)			67.9 (1.6)	−4.3			0.1
Age group															
15–44	93.4 (2.5)			91.4 (3.1)	+2.0		0.7	78.4 (4.4)			75.9 (4.6)	+2.5			0.7
45–54	91.5 (1.6)			95.1 (1.2)	−3.6		0.1	72.9 (2.7)			83.1 (2.1)	−10.2			0.007
55–64	85.9 (1.3)			88.7 (1.1)	−2.8		0.2	64.9 (1.9)			71.8 (1.8)	−6.9			0.009
65–74	81.7 (1.2)			83.2 (1.3)	−1.5		0.6	59.4 (1.9)			64.6 (1.9)	−5.2			0.08
75+	65.1 (1.9)			69.3 (1.6)	−4.2		0.8	42.6 (3.1)			49.0 (2.4)	−6.4			0.4
Males															
15–44	95.7 (2.5)			90.3 (4.3)	+2.4		0.3	78.2 (5.6)			72.0 (6.0)	+6.2			0.4
45–54	89.9 (2.1)			94.5 (1.7)	−4.6		0.09	68.8 (3.7)			79.4 (2.8)	−10.6			0.04
55–64	84.8 (1.7)			86.9 (1.5)	−2.1		0.4	59.1 (2.5)			70.1 (2.2)	−11.0			0.005
65–74	79.2 (1.6)			81.5 (1.7)	−2.3		0.5	56.7 (2.6)			61.7 (2.5)	−5.0			0.2
75+	62.1 (2.7)			69.2 (2.3)	−7.1		0.4	44.6 (4.9)			49.7 (3.5)	−5.1			0.3
Females															
15–44	88.0 (5.9)			92.3 (4.7)	−4.3		0.6	77.5 (7.5)			81.5 (7.1)	−4.0			0.6
45–54	94.9 (2.2)			96.2 (1.8)	−1.3		0.6	79.3 (4.0)			89.9 (2.9)	−10.6			0.05
55–64	87.9 (2.0)			91.7 (1.6)	−3.8		0.2	74.1 (2.8)			74.5 (2.9)	−0.4			0.6
65–74	85.8 (1.7)			86.1 (2.0)	−0.3		0.9	63.8 (2.9)			69.5 (2.9)	−5.7			0.3
75+	68.3 (2.6)			69.4 (2.3)	−1.1		0.6	42.8 (4.1)			48.6 (3.2)	−5.8			0.8

*RS* relative survival, *SE* standard error, *p (country) p* value for comparison between the USA and Germany

^a^Age adjusted

Because CLL is not curable but has a long disease course with a high percentage of 5-year survivors, 10-year survival was also calculated. Ten-year relative survival for the years 2009–2011 was 59.5 % for Germany and 64.7 % for the USA (Table 3). Age-specific analysis showed lower 10-year survival for Germany at all ages except for age 15–44, with the largest differences being observed for ages 45–54 and 55–64 (−10.2 and −6.9 % units, respectively) (Table 3). Analysis by gender showed a significantly lower survival for men in Germany at 57.4 versus 63.0 % in the USA (*p* = 0.0001) and a slightly smaller, not statistically significant difference for women with survival estimates of 63.6 and 67.9 %, respectively, in Germany and the USA. When gender- and age-specific analyses were performed, survival was lower in Germany for each age group and both genders with the exception of men age 15–44. The largest differences were for men at age 55–64 at −11.0 % units (*p* = 0.005) and men and women age 45–54 at −10.6 % units for both (*p* = 0.04 for men, *p* = 0.05 for women).

**Fig. 1:**
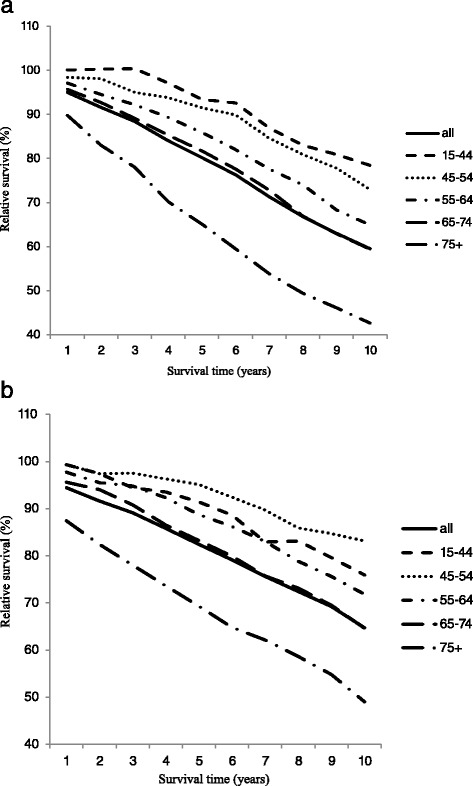
Ten-year relative survival for patients with CLL in 2009–2011 in **a** Germany and **b** the USA by age group: overall (*solid line*), 15–44 (*dashed line*), 45–54 (*dotted line*), 55–64 (*dot/dash line*), 65–74 (*long dash line*), and 75+ (*long dash/dot line*)

When survival by year after diagnosis was examined, there was a nearly linear decrease from 1- to 10-year relative survival in both Germany and the USA (Fig. 1). It is notable that a plateau in survival was not observed in either Germany or the USA in any age group or at any time after diagnosis, demonstrating that long-term survivors are not cured but continue to be at risk from the disease. Survival decreased with age for patients diagnosed in Germany, but in the USA, survival was actually consistently better for patients age 45–54 than for ages 15–44.

## Discussion

Overall, 5-year relative survival has reached levels of greater than 75 % or higher in CLL patients in Germany and the USA. Survival estimates varied relatively little between the two countries, although a statistically significant difference was observed for men and for all patients age 45–54 concerning 10-year relative survival. Although survival decreases with age, the decrease is not as abrupt as that seen in some other hematologic malignancies, with survival estimates for ages 45–54 being fairly similar to those for ages 15–44. Five-year relative survival increased only slightly in the calendar periods examined. Ten-year survival was lower than 5-year survival, probably indicating ongoing mortality due to CLL, although due to the nature of the analysis, 10-year survival estimates included older data than 5-year survival estimates. Women had a higher survival estimate in both countries, consistent with prior studies which have shown longer survival and better response to therapy in women [20].

CLL is a heterogeneous disease with variable presentation. Appropriate therapy can include observation, chemotherapy, chemoimmunotherapy, or hematopoietic stem cell transplant, depending on disease and patient characteristics. Survival for patients with CLL has been documented to have increased since the late twentieth century in most population-based studies [3, 21, 22], although others have found no major difference in survival during similar time periods [23]. Our current data suggest that there continues to be incremental improvement in survival for patients with CLL, which was most pronounced among patients age 45–54 in the period studied. Interestingly, the increase in survival for patients age 45–54 between 2003–2005 and 2009–2011 made this population’s relative survival more similar to that of the next younger age group.

Changes in survival in CLL may be due to better understanding of prognosis, leading to more appropriate therapy, or changes in therapy. Classic prognostic factors in CLL include clinical factors such as Rai stage, age, gender, and comorbid conditions as well as laboratory factors such as lymphocyte doubling time [24]. Additional major prognostic indicators include CD38 expression, presence or absence of mutation in the variable heavy immunoglobin (IgVH), and specific cytogenetic abnormalities [25]. Both CD38 expression and IgVH mutation status were reported as important clinical markers in 1999 [26, 27]. ZAP70 expression has been used as an easier-to-measure proxy for IgVH mutational status [28]. Specific chromosomal abnormalities associated with poor outcomes have been reported since 2000 as well [29]. Other markers of poor prognosis have been proposed [30–32], and thus further refinement of prognostic stratification may be expected to continue.

In terms of treatment, several changes have occurred in the early twenty-first century. Starting in about the year 2000, several clinical trials demonstrated better response and progression-free survival for high risk patients treated with fludarabine versus alkylating agents [33, 34]. Although the monoclonal antibody rituximab is not an effective therapy for CLL as monotherapy, its introduction into combination chemotherapy regimens along with fludarabine and possibly an alkylating agent has led to improved survival for younger and fit patients (though it may be too toxic for older or less fit patients) [35]. Bendamustine is a highly active agent in CLL as well, with response rates approaching 90 % in clinical trials [36]. It has been used for longer in Germany than in the USA but has become one frequently used option in both countries. A trial directly comparing bendamustine and rituximab versus fludarabine, an alkylating agent, and rituximab was recently completed [37], and initial results suggest fludarabine, cyclophosphamide, and rituximab (FCR) may be superior for fit patients without p17-, but not in older patients or those with more comorbidities. Other agents and combinations of these agents with fludarabine, rituximab, or other agents have increased the options for treating patients with CLL as well [20]. Very recent clinical trials of newer agents such as idelalisib and ibrutinib have shown dramatic improvements in survival in patients with CLL in those trials [38, 39], but the results of these new treatment options will not be evident on the population level for some time. However, data from clinical trials suggest that these and similar agents may substantially alter the natural history of the disease [40]. In addition, they are well tolerated and can be given to patients with poor performance status who make up a large proportion of CLL patients. Thus, current survival expectations for patients with CLL may be underestimated by our study due to changes in treatment that have occurred in the last 3 years and are therefore not yet available in the population level databases. Finally, stem cell transplant is an option for fit patients with poor prognosis disease and otherwise good clinical status [41]. Stem cell transplantation offers the only potential for cure in patients with CLL but is used only in a minority of cases because of the risk of early mortality and morbidity due to the treatment and the relatively low risk nature of many CLL clones.

Strengths of our study include the use of large databases with survival information from a number of regions in each country sampled, allowing for evaluation of survival and subgroup analysis of this relatively rare leukemia and decreasing the risk that survival differences between individual areas in each country will unduly influence the impression of survival in that country overall. Use of population level data provides a more accurate understanding of how probable it is for a patient with a given disease in the general population to survive in contrast to data provided by clinical trials which tend to present the best-case scenario with respect to survival (i.e., only patients with good performance status and few or no comorbidities are generally eligible for clinical trial participation) and may not be realistic for the general population [42]. Additionally, the use of period analysis and modelled period analysis provides the most up-to-date estimates of possible survival [14, 18].

In interpreting our results, several limitations should also be considered. First, despite the large databases, the relative rarity of CLL limits our ability to detect minor differences in survival, especially for younger patients in whom the condition is rarer. Second, in the absence of a national death index in Germany, most cancer registries rely on record linkage with vital statistics from the state that they cover and may miss deaths among patients who move out of the state. Nevertheless, previous validation studies have suggested potential overestimation of survival due to deaths missed by migration to be very small [43]. In the USA, deaths are derived from the National Death Index, and therefore this is less of a problem, though the possibility of missing deaths because patients move out of the country is a potential concern. Third, information on treatment, particularly chemotherapy, and disease biology are very limited or absent in both databases, and therefore we cannot definitely establish the extent to which treatment and differences in disease biology (i.e., frequency of heavy chain rearrangement or chromosomal abnormalities) affect observed differences in survival or changes in survival over time. Fourth, there is some evidence that survival estimates from the SEER database may be higher than survival in the US population in general [11], so some caution is necessary when comparing survival in the two countries. Fifth, registry samples in Germany have changed over time, i.e., some registries started later, and therefore the underlying population is not entirely consistent throughout the study period. Sixth, changes in the diagnostic criteria of CLL made in 2008 requiring a B-cell count of greater than 5000 [1] may have subtly changed the population diagnosed, resulting in later diagnosis of CLL and therefore a shorter “patient career” for patients diagnosed after that time, potentially obscuring some increases in survival in the later time period. However, any changes due to this issue would be present in both countries.

Finally, several of the newer German registries are still in the build-up phase and the proportion of cases identified by DCO is still relatively high in some of these registries, especially at earlier time points. Exclusion of these DCO cases in the analysis may have led to some overestimation of survival in Germany. As a result, the survival gap between the USA and Germany may have been underestimated to some extent [43, 44].

## Conclusions

In summary, high 5-year relative survival for patients with CLL in Germany and the USA are meanwhile observed on the population level, exceeding 90 % in patients age 54 or younger and over 75 % overall. Ongoing increases in survival were observed overall for Germany and in the USA in the first decade of the twenty-first century. Further research into ways to increase survival for older CLL patients are needed to reduce the persistent large age-related survival disparity.
